# Towards precise PICO extraction from abstracts of randomized controlled trials using a section-specific learning approach

**DOI:** 10.1093/bioinformatics/btad542

**Published:** 2023-09-05

**Authors:** Yan Hu, Vipina K Keloth, Kalpana Raja, Yong Chen, Hua Xu

**Affiliations:** School of Biomedical Informatics, University of Texas Health Science Center at Houston, Houston, TX 77054, United States; Section of Biomedical Informatics and Data Science, School of Medicine, Yale University, 100 College St, New Haven, CT 06510, United States; Section of Biomedical Informatics and Data Science, School of Medicine, Yale University, 100 College St, New Haven, CT 06510, United States; Center for Health Analytics and Synthesis of Evidence (CHASE), Department of Biostatistics, Epide-miology and Informatics, University of Pennsylvania, 423 Guardian Dr, Philadelphia, PA 19104, United States; Penn Medicine Center for Evidence-based Practice (CEP), University of Pennsylvania, 3600 Civic Center Blvd, Philadelphia, PA 19104, United States; Section of Biomedical Informatics and Data Science, School of Medicine, Yale University, 100 College St, New Haven, CT 06510, United States

## Abstract

**Motivation:**

Automated extraction of population, intervention, comparison/control, and outcome (PICO) from the randomized controlled trial (RCT) abstracts is important for evidence synthesis. Previous studies have demonstrated the feasibility of applying natural language processing (NLP) for PICO extraction. However, the performance is not optimal due to the complexity of PICO information in RCT abstracts and the challenges involved in their annotation.

**Results:**

We propose a two-step NLP pipeline to extract PICO elements from RCT abstracts: (i) sentence classification using a prompt-based learning model and (ii) PICO extraction using a named entity recognition (NER) model. First, the sentences in abstracts were categorized into four sections namely background, methods, results, and conclusions. Next, the NER model was applied to extract the PICO elements from the sentences within the title and methods sections that include >96% of PICO information. We evaluated our proposed NLP pipeline on three datasets, the EBM-NLP*_mod_* dataset, a randomly selected and re-annotated dataset of 500 RCT abstracts from the EBM-NLP corpus, a dataset of 150 Coronavirus Disease 2019 (COVID-19) RCT abstracts, and a dataset of 150 Alzheimer’s disease (AD) RCT abstracts. The end-to-end evaluation reveals that our proposed approach achieved an overall micro F1 score of 0.833 on the EBM-NLP*_mod_* dataset, 0.928 on the COVID-19 dataset, and 0.899 on the AD dataset when measured at the token-level and an overall micro F1 score of 0.712 on EBM-NLP*_mod_* dataset, 0.850 on the COVID-19 dataset, and 0.805 on the AD dataset when measured at the entity-level.

**Availability and implementation:**

Our codes and datasets are publicly available at https://github.com/BIDS-Xu-Lab/section_specific_annotation_of_PICO.

## 1 Introduction

Healthcare providers rely on the evidence from published biomedical literature for assessing the effectiveness of a new treatment or intervention for diseases. With the evidence and insights gained from well-designed clinical studies such as randomized control trials (RCTs) in PubMed (Frost *et al.* 2020, Abani *et al.* 2021, Emani *et al.* 2021), it is possible to explore the best treatment options for diseases such as Coronavirus Disease 2019 (COVID-19) (Emani *et al.* 2021). However, the number of RCTs that keep growing every day has become a challenge for clinicians to keep themselves up to date with the new evidence. In 2020, a total of 29 256 RCT abstracts (80 RCT abstracts per day) were added to PubMed. This increased to 29 983 RCT abstracts (82 RCT abstracts per day) in 2021 and 28 482 RCT abstracts (78 RCT abstracts per day) in 2022. These estimates show a continuously growing number of publications related to RCT and, it has become extremely difficult for clinicians to gain knowledge from these publications to provide the best care for their patients. Currently, more than 595 000 RCT abstracts are in PubMed.

In 1995, the PICO (P: Population, I: Intervention, C: Comparison/Control, O: Outcomes) framework (Richardson *et al.* 1995) was introduced to formulate a well-defined research question and facilitate the literature search for evidence-based medicine (EBM). Manual synthesis of PICO-based evidence from the published literature is both time-consuming and costly. Hence, automated approaches were developed for extracting PICO elements. Earlier works were based on rule-based approaches and machine learning (ML) approaches (e.g. Support Vector Machine, Random Forest, Conditional Random Field) and rely heavily on hand-crafted features (Huang *et al.* 2006, Demner-Fushman and Lin 2007, Boudin *et al.* 2010, Chabou and Iglewski 2015, 2018). Recently, deep learning-based (DL) approaches are applied to overcome the tedious feature engineering task and boost performance (Jin and Szolovits 2018b, Zhang *et al.* 2020). Both ML/DL approaches rely heavily on the annotated corpus to achieve high performance. The time and cost involved in the manual annotation by domain experts, the distribution of PICO elements within RCT abstracts, and the variations in PubMed abstracts (e.g. structured and unstructured) are the major reasons for the lack of a large, publicly available annotated corpus for PICO extraction.

Nye *et al.* (2018) released the EBM-NLP corpus with 4993 RCT abstracts annotated (using crowdsourcing) with PICO elements at two levels of granularity. The first level is the named span-level where the phrases containing Population, Intervention, and Outcome (PIO) information are annotated. The second level further distinguishes the named span-level with more fine-grained labels (e.g. distinguish P according to gender, age, condition, etc.). Though the EBM-NLP corpus has revived the PICO extraction task, it has certain limitations. The overall named entity recognition (NER) model performance is not satisfying (a token-level F1 score of 68% on the span-level and 48% on the hierarchical labels). There is no entity-level evaluation or information retrieval evaluation at the abstract level. Based on our observation, probable reasons for low performance are: (i) Information regarding PICO elements is scattered throughout the abstract with interventions and outcomes often presented multiple times with different lexical variants. (ii) PICO elements in RCTs can be complex, making the annotation task complex. For example, all interventions may not refer to the clinical trial under consideration (e.g., mentions of interventions of prior clinical trials), and identifying distinct outcomes of a particular intervention in multi-arm trials is often difficult. (iii) PICO elements can vary widely across different diseases hence requiring large datasets to learn meaningful patterns. For example, intervention may be a pharmacologic substance (e.g. doxorubicin) for diseases such as cancer or a music-based therapy for certain neurological disorders. (iv) The EBM-NLP corpus utilized a crowdsourcing approach for the annotation process. The expert annotation is limited to only 200 abstracts. The remaining abstracts in the corpus were annotated by the annotators recruited by Amazon Mechanical Turk (AMT), who were not well-trained. The inter-annotator agreement (IAA) is low (0.50 for P, 0.59 for I, and 0.51 for O) even among the medical experts. The annotation schema is also complex, and it is difficult for the annotators to comprehend all the details. This results in annotation inconsistencies.

We hypothesized that selecting only the specific sections of an RCT abstract and title that contain relevant PICO information for annotation can significantly reduce the annotation complexity, effort, and the issues mentioned above. In the current study, we analyzed the distribution of PICO elements in different sections of RCT abstracts. Our findings suggested that the Title and Methods section covers most of the PICO elements. Consequently, we developed a two-step natural language processing (NLP) pipeline to identify and extract the PICO elements. To assess its generalizability, we further evaluated it on three different datasets namely EBM-NLP_*mod*,_ dataset, Alzheimer’s disease (AD) dataset, and COVID-19 dataset. The major contributions of this work are:

1. A novel two-step NLP pipeline that classifies the sentences from a PubMed abstract into different sections, backgrounds, methods, results, and conclusions and extracts the PICO elements.
2. EBM-NLP_*mod*_ dataset derived from the EBM-NLP corpus. The dataset includes 500 randomly selected RCT abstracts. The PICO elements were re-annotated to overcome the limitations of the EBM-NLP corpus.
3. Two additional disease-specific datasets related to AD and COVID-19. Each dataset includes 150 RCT abstracts annotated with PICO elements.
4. A simplified annotation guideline for annotating all P, I, C, and O elements at a single level compared to multi-level annotation and annotating C as part of I by Nye *et al.* (2018). Such an annotation can reduce the annotation complexity and inconsistencies. Our guidelines and datasets are publicly available at https://github.com/BIDS-Xu-Lab/section_specific_annotation_of_PICO

## 2 Related work

### 2.1 Classification of sentences from PubMed abstracts

The clinicians use PICO for querying evidence for improving patient care. Manual extraction of PICO information from a huge volume of PubMed abstracts is impossible. This motivated the research community to develop automated approaches for PICO extraction. An earlier study by Demner-Fushman and Lin (2007) used MetaMap, hand-written rules, and statistically derived features for building a classifier for extracting the population, intervention, and outcome elements. Certain approaches classify the sentences into general categories such as Aim and Method (McKnight and Srinivasan 2003, Xu *et al.* 2006, Ruch *et al.* 2007) and extract the PICO elements (Chung 2009, Kim *et al.* 2011). A wide range of machine learning algorithms including Naïve Bayes (Ruch *et al.* 2007), Support Vector Machines (McKnight and Srinivasan 2003, Shimbo *et al.* 2003), Hidden Markov Models (Lin *et al.* 2006, Xu *et al.* 2006), and Conditional Random Fields (CRF) (Chung and Coiera 2007, Hirohata *et al.* 2008, Chung 2009, Kim *et al.* 2011) were explored for sentence classification. Kim *et al.* (2011) constructed a corpus of 1000 abstracts called NICTA-PIBOSO corpus, by manually annotating the sentences into six categories namely background, population, intervention, outcome, study design, and others. The authors used CRF for sentence classification and the model learned the information from the lexical, semantic, structural, and sequential features.

Dernoncourt *et al.* (2016) presented a neural network architecture that learned the features and token embeddings jointly, and predicted the classes for all sentences in the RCT abstracts. Dernoncourt and Lee (2017) created a corpus called PubMed 200k RCT corpus. The corpus contains 200 000 RCT abstracts from PubMed and includes five annotation labels namely objectives, background, methods, results, and conclusions for classifying the sentences. Jin and Szolovits (2018a,b) treated PICO detection as a sequential sentence classification task rather than a single sentence classification task. They utilized neural network architectures such as long short-term memory (LSTM) (Jin and Szolovits 2018b) and bi-directional long short-term memory (Bi-LSTM) (Jin and Szolovits 2018a) to encode the contextual content from the preceding and succeeding sentences to improve the prediction of the current sentence. Recently, several deep learning approaches that utilize pre-trained language models such as SciBERT and BERT are applied to improve the performance of sentence classification (Cohan *et al.* 2019, Yamada *et al.* 2020, Shang *et al.* 2021).

### 2.2 Recognition of PICO elements

NER is used to identify the PICO elements (Nguyen *et al.* 2017, Nye *et al.* 2018, Kang *et al.* 2019, 2021, Zhang *et al.* 2020, Dhrangadhariya *et al.* 2021, Liu *et al.* 2021b). Nye *et al.* (2018) presented two baseline models, the linear CRF model and the LSTM-CRF model for identifying PICO elements in the EBM-NLP corpus. A recent study showed improved performance on PICO extraction when the NER model was first pre-trained on the EBM-NLP corpus, and further fine-tuned with the additional data annotated by themselves (Kang *et al.* 2019). Zhang *et al.* (2020) proposed an approach by combining sentence classification, disease entity recognition, and disease mapping using various deep learning models (convolutional neural network, Bi-LSTM, etc.) for extracting P and O elements. To alleviate the reliance on time-consuming manual annotation by experts, a span detection approach for PICO extraction that uses only low-quality, crowd-sourced, sentence-level annotations as inputs, was proposed by Liu *et al.* (2021b). The authors applied a masked span prediction task in which input spans were replaced with predefined mask tokens and a pre-trained neural language model [BLUE (Peng *et al.* 2019)] was used to infer which spans contribute most to the PICO sentence classification results using the EBM-NLP corpus. A multi-task learning approach that learns and recognizes both coarse-grained descriptions (e.g. 40 children aged 7–11 with autism spectrum disorder) and constituent finer semantic units (e.g., “40” shows “sample size”, “7–11” shows “age” and “autism spectrum disorder” shows “condition”) was explored by Dhrangadhariya *et al.* (2021). In that study, the EBM-NLP corpus was utilized as it provided a multi-level annotation: the span-level (level 1) annotation corresponds to the coarse-grained descriptions and other levels of annotation focus on specific semantic units. Recently, the Easy Data Augmentation (Wei and Zou 2019) technique incorporated with the Unified Medical Language System (UMLS) knowledge (including synonym replacement, random insertion, random swap, and random deletion) was evaluated on PICO extraction (Kang *et al.* 2021).

## 3 Materials and methods

**Figure 1.**
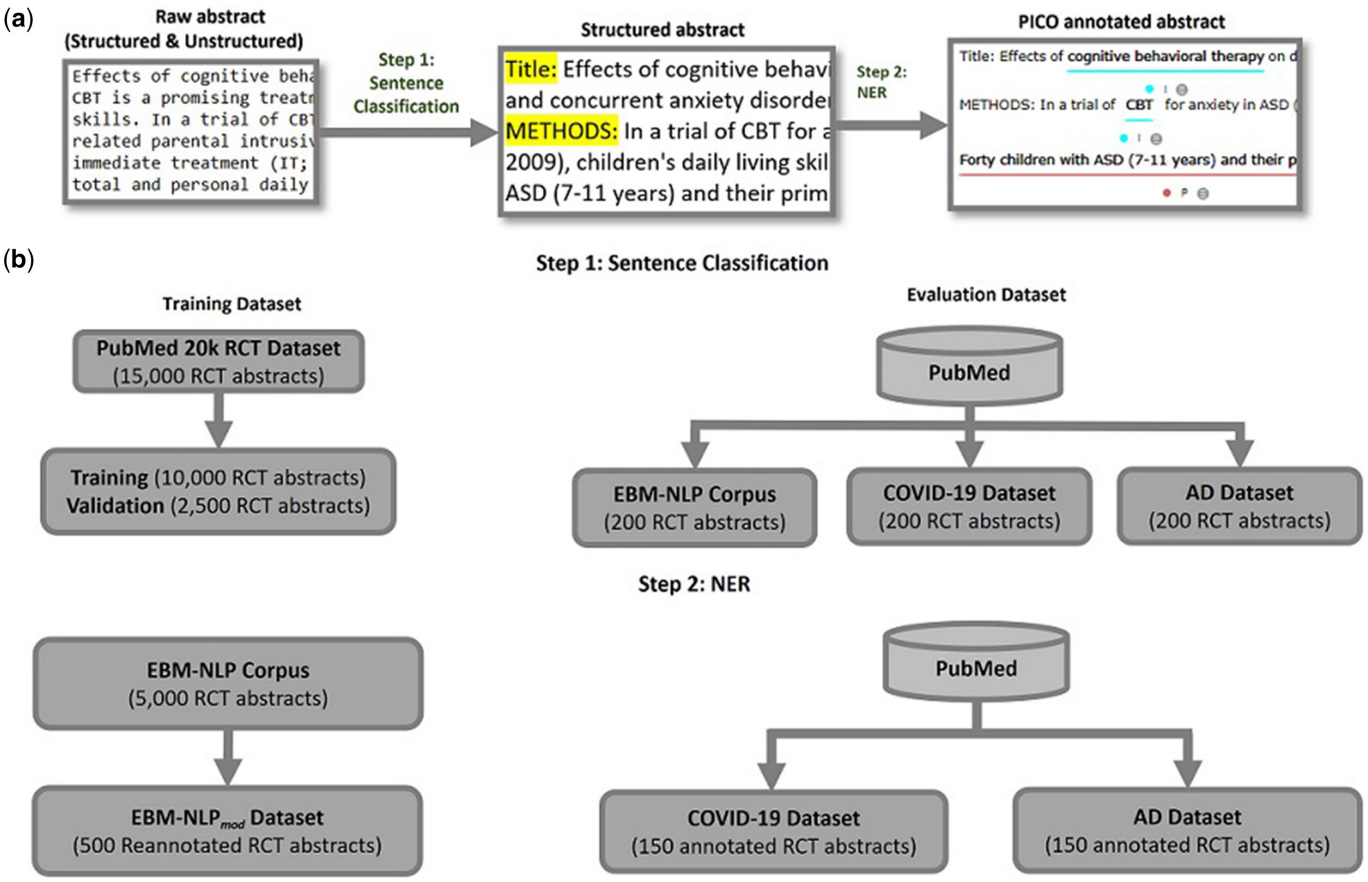
Overview of PICO extraction system. (a) The two-step PICO extraction system that includes a sentence classification and NER of PICO elements. (b) Training and test datasets for sentence classification and NER

The overview of our NLP pipeline is illustrated in Fig. 1. First, the sentences in both structured and unstructured RCT abstracts were classified into background, methods, results, and conclusions using our recent work on sentence classification (Hu *et al.* 2022). Next, P, I, C, and O elements were extracted using a NER model.

### 3.1 Distribution of PICO elements in RCT abstracts

We conducted a preliminary evaluation of the distribution of PICO elements across different sections of the RCT abstract. We randomly selected 30 RCT abstracts and reviewed them manually to identify the unique mentions of PICO elements in different sections of the abstracts. The purpose was to identify the specific sections with high coverage of PICO elements. We hypothesize that annotating only these sections may reduce the redundancy, ambiguity, and time and effort involved in the manual annotation. We also hypothesize that our proposed approach is liable only for a minimum loss of information. Titles of RCT abstracts usually include P and I elements. They provide a precise, and accurate description of the study, and are easy to annotate. Our analysis shows that the Methods section achieved the highest coverage of 95.2% among all the sections. The Title and Methods sections together achieve coverage of 96.7% (Table 1). The PICO elements in the results section are often duplicates of the methods section. Thus, we considered only the Title and Methods sections for annotation. The annotation can easily be extended to other sections with our sentence classification model.

**Table 1.** Distribution of PICO entities in different sections of the abstract for a random selection of 30 abstracts.

Entity	TT^a^	BK^a^	MT^a^	RS^a^	CC^a^	Total
P	30	30	28	5	26	30
I	30	30	30	30	30	30
C	5	0	10	11	8	12
O	6	4	72	71	10	75
Coverage	0.483	0.435	0.952	0.800	0.503	

a TT, Title; BG, Background; MT, Methods; RS, Results; CC, Conclusion.

### 3.2 Datasets

#### 3.2.1 Sentence classification

##### 3.2.1.1 Training data

For training the sentence classification model, we utilized the PubMed 20k RCT dataset (Dernoncourt and Lee 2017) which includes 15 000 PubMed abstracts for training, 2500 abstracts for validation, and 2500 abstracts for testing. We used the training data for model training, and the validation data for parameter tuning. There are about 240k sentences in the dataset and these sentences belong to one of the following section labels: background, objective, methods, results, or conclusions. While our previous work (Hu *et al.* 2022) shows high performance in predicting methods, results, and conclusion sections, the classifier has great difficulty distinguishing the background and objective sections. The difference between the sentences from the background and objective sections is less obvious when compared to other sections. We relabeled the sentences from the objective section as background and redefined the section labels as background, methods, results, and conclusions.

##### 3.2.1.2 Evaluation data

We created three datasets to evaluate and report the performance of the sentence classification model. We randomly selected 200 structured RCT abstracts from the EBM-NLP corpus, 200 structured RCT abstracts from the COVID-19 dataset, and 200 structured RCT abstracts from the AD dataset. The COVID dataset and AD dataset were retrieved from PubMed using the Boolean query, “COVID-19”[Mesh] and “Alzheimer Disease”[Mesh] with publication type limited to Randomized Controlled Trial between 09/01/2021 and 11/05/2021. Following the methodology described by Dernoncourt and Lee (2017) on constructing the PubMed 200k RCT dataset, we used the NLM Label List and Category Mappings (available at: https://lhncbc.nlm.nih.gov/ii/areas/structured-abstracts.html) to tag each sentence with a section label (i.e. background, methods, results, and conclusions). The number of sentences in each label across the three datasets is shown in Table 2.

**Table 2.** Number of sentences in each class for the sentence classification evaluation dataset.

	BG^a^	MT^a^	RS^a^	CC^a^	Total
EBM-NLP^b^	430	747	817	388	2382
COVID-19	627	1114	876	356	2973
AD	486	805	735	363	2389
Total	1543	1543	1543	1543	7744

a BG, Background; MT, Methods; RS, Results; CC, Conclusions.

b Two-hundred randomly selected structured abstracts from the original EBM-NLP corpus.

#### 3.2.2 Named entity recognition

We derived the EBM-NLP_*mod*_ dataset from the EBM-NLP corpus by randomly selecting 500 RCT abstracts. The PICO elements in these abstracts were re-annotated according to our annotation guidelines (see Section 3.3). We created two disease-specific datasets, one for COVID-19 and the other for AD to evaluate the performance of our NLP pipeline on PICO extraction. We retrieved 552 RCT abstracts for COVID-19 (access date, 09/01/2021) and 1980 RCT abstracts for AD (access date, 11/05/2021) from PubMed. We randomly chose 150 RCT abstracts for COVID-19 and 150 RCT abstracts for AD and annotated them to train and evaluate the NER models.

In our dataset splits, we have meticulously ensured that the test set used for the NER task does not overlap with the training data for sentence classification. Our approach eliminated potential data leakage and ensured an unbiased evaluation of both tasks.

### 3.3 Section-specific annotation

Annotating a high-quality dataset is a labor-intensive task and identifying the domain experts is challenging. Though the crowdsourcing approaches have shown some promising results on corpus generation, the IAA (e.g. EBM-NLP corpus) is relatively low. This results in a suboptimal performance of a NER model.

Initially, we followed the annotation guidelines from EBM-NLP to annotate the PICO elements. The IAA reported using Cohen’s Kappa coefficient was only 0.3 (Supplementary Materials S1.2 for the equation for Cohen’s Kappa coefficient). Further investigation revealed three major reasons for achieving a low kappa coefficient: (i) the original annotation guidelines from EBM-NLP are complex and complicated. It first necessitates annotating the Participant, Intervention, and Outcome elements at the span-level, and further annotates the specific details at a granular level. For instance, Participant is annotated at the span-level and the specific details about the participant (i.e. age, gender, and condition) are annotated at the granular level. Likewise, Intervention is annotated at the span-level and the specific details about the intervention (i.e. physical, non-physical, and Control) are annotated at the granular level. Note that Control is annotated as a subtype of Intervention. (ii) The original annotation guidelines lack specific rules for defining entity boundaries for the PICO elements. In addition to defining the PICO elements with examples of inclusion and exclusion, the guidelines only mention - “mark the longest contiguous text that includes such a description.” While differences in span boundaries in annotations by different annotators are a major reason behind low IAA, our experience in annotating the documents for other studies has shown that the specific rules regarding modifiers, articles, prepositions, and overlapping entities improve the IAA. (iii) The repeated mentions of interventions and outcomes across different sections of the abstract lead to ambiguity and missing annotations, especially in complex interventions and multi-arm trials. For example, consider a study comparing a multicomponent community health program versus usual care on several health outcomes. As we move along different sections of the abstract, we may come across repeated mentions of these interventions including a detailed mention, a specific component mention, abbreviation of the program, and even some generic reference such as “the program.” Additionally, mentioning the interventions that are not pertinent to the current trial further confuses the annotators (Nye *et al.* 2018).

We resolve the issues observed in the original annotation guidelines, by: (i) annotating the PICO elements at a single level; (ii) enriching the annotation guidelines with a set of linguistic rules to define the boundaries (see Supplementary Materials S3), and (iii) retaining the sentences only from the Title and Methods sections (see Supplementary Materials S3). This is based on our preliminary experiments as shown in Table 1. Annotating the PICO elements mentioned in the Title and Methods sections took only 60 s per abstract. This is significantly lower than the time required to annotate the PICO elements mentioned in all the sections of a PubMed abstract (i.e. 146 s). Switching from the hierarchical annotation to a single-level annotation was based on several limitations and challenges that we noticed in the original multi-level annotation. Firstly, the hierarchical-level annotation significantly increases the time, effort, and cost of the annotation process. Secondly, prior research using the EBM-NLP corpus has reported issues with the fine-grained classification of PICO elements utilized for the hierarchical annotation. One such issue was concerned with the “Intervention” element. According to Dhrangadhariya *et al.* (2021), even the human annotators find it difficult to classify certain interventions as education or psychological. Their experiments showed the least performance of 0.31 F1-score on the “Intervention” class. From the error analysis, the authors suspect that the ambiguities arising from the split of coarse-grained PICO into fine-grained PICO classes were one of the issues for such low performance.

Using the revised annotation guidelines, two annotators with medical background re-annotated the EBM-NLP_*mod*_ dataset and annotated two additional datasets related to COVID-19 and AD. The IAA was calculated for each PICO element and the entire dataset using Cohen’s Kappa coefficient. The approach achieved the Cohen’s Kappa coefficient of 0.714, 0.808, 0.701, and 0.790 for the P, I, C, and O components, respectively, and 0.746 for all PICO elements.

### 3.4 Prompt-based learning for sentence classification

Recent works show the feasibility of using natural language prompts for tuning the pre-trained language models for specific downstream tasks (Petroni *et al.* 2019, Ding *et al.* 2021, Liu *et al.* 2021a). In our prior research (Hu *et al.* 2022), we applied prompt-based learning to classify sentences from RCT abstracts. In brief, our approach classifies sentences by predicting the mask position in RCT abstracts using prompt-based learning. Other existing approaches use traditional machine learning and deep learning to classify sentences. The performance of our sentence classification approach surpasses the performance of the previous state-of-the-art approach using Hierarchical Sequential Labeling Network (HSLN) (Jin and Szolovits 2018a). A more detailed description of our method is in Supplementary Materials S1.1. We applied the model from our previous work to classify the sentences in the RCT abstracts from the EBM-NLP_*mod*_ dataset, COVID-19 dataset, and AD dataset. The parameters used in our prompt-based learning approach were set as follows: dropout = 0.5, batch size = 8, learning rate = 6e−6, optimizer = AdamW, and learning rate decay = 0.01. We evaluated our prompt-learning model and compared our model with that of the HSLN architecture on the EBM-NLP_*mod*_ dataset, COVID-19 dataset, and AD dataset independently. We used the standard evaluation metrics, precision (P), Recall (R), and F1 scores.

### 3.5 NER for PICO extraction

A recent work on identifying P, I, and O elements using LSTM-CRF within BERT achieved a 0.68 F1-score on the EBM-NLP corpus when evaluated at the token-level. Another work by Gu *et al.* (2022) used the pre-trained model from PubMedBERT and achieved a 0.73 F1 score on the same corpus at the same token-level. We used the same experimental setting (i.e. data, training parameters) and evaluation script provided by the EBM-NLP corpus and reported the performance at the token-level using micro-averaged precision, recall, and F1-score (Supplementary Results Table S3). In addition, we also reported the performance at entity-level by matching the exact spans.

We trained the NER models for the EBM-NLP_*mod*_ dataset, COVID-19 dataset, and AD dataset using PubMedBERT for five epochs with a learning rate of 1e−5 and a batch size of 32. We also experimented with the NER models with other pre-trained models including BERT (Devlin *et al.* 2018), BioBERT (Lee *et al.* 2020), BioM-ALBERT (Alrowili and Vijay-Shanker 2021), and BioM-ELECTRA (Gu *et al.* 2022). The PubMedBERT was better among all pre-trained models in our previous study.

We performed five-fold cross-validation and calculated the average micro precision, recall, and F-1 scores. We also evaluated the performance of each PICO element. For all datasets, we used one-fold as the development set for tuning the hyperparameters and tested a range of values: learning rate (1e−5, 3e−5, 4e−5, 5e−5, 6e−5), batch size (8, 16, 32), and epoch number (5–20). The model achieved the best performance when learning rate = 5e−5, batch size = 8, and epoch number = 10.

### 3.6 Evaluation

#### 3.6.1 NER evaluation

We evaluated the performance of the NER models on two levels: (i) token-level; and (ii) entity-level. For the token-level evaluation, we used the original evaluation script from EBM-NLP corpus. The script excludes all the “Outside” labels in Inside, Outside, Beginning (IOB) tagging for a fair comparison. The token-level evaluation may not be the best approach because many biomedical named entities include multiple tokens, and the goal is to identify the whole entity. For example, the Participant element, “86 hospitalized COVID-19 patients,” was partially identified as “86 hospitalized” and “patients” in the token-level evaluation, The condition, “COVID-19” was omitted. This results in an incomplete representation of the Participant element. For the entity-level evaluation, the entire entity span is viewed as the Participant element. The evaluation preserves comprehensive information. The entity-level evaluation is more reliable than the token-level evaluation. For both types of evaluations, we calculated precision (P), recall (R), and F1 scores for each PICO element, as well as the micro-averaged overall scores for P, R, and F1. These micro-averaged overall scores are computed using the sum of True Positives (TP), False Positives (FP), and False Negatives (FN) from each PICO element. The formula for these scores is provided in Supplementary Materials S1.3.

#### 3.6.2 End-to-end evaluation

In addition to evaluating the sentence classification model and the NER system individually, we also performed an end-to-end evaluation to assess the combined performance of the sentence classification model and NER. For a more direct comparison with the standalone NER module, we implemented our two-step pipeline on the identical dataset used to evaluate the standalone NER module. We maintained a consistent evaluation metric used for the standalone NER module.

## 4 Results

### 4.1 Performance of the sentence classification module

Our approach achieved an overall F1 score of 0.953 on the EBM-NLP_*mod*_ dataset, 0.931 on the COVID-19 dataset and 0.962 on the AD dataset. The F1 score for the *Methods* section alone is 0.949 for the EBM-NLP_*mod*_ dataset, 0.923 for the COVID-19 dataset, and 0.955 for the AD dataset (Table 3). A comparison between our sentence classification approach and the existing state-of-the-art approach using HSLN architecture validates the superior performance of our approach. A detailed comparison of both approaches and their performance is in Supplementary Results Table S4.

**Table 3.** Performance of the prompt-based sentence classification model on the evaluation dataset of COVID-19, AD, and EBM-NLP_*mod*_.

	EBM-NLP_*mod*_	COVID-19	AD
Class	P/R/F1	P/R/F1	P/R/F1
BG	0.911/0.977/0.943	0.897/0.975/0.934	0.934/0.988/0.960
MT	0.946/0.952/0.949	0.913/0.933/0.923	0.956/0.953/0.955
RS	0.967/0.961/0.964	0.977/0.887/0.930	0.987/0.948/0.967
CC	0.989/0.910/0.948	0.947/0.955/0.951	0.965/0.975/0.970
Overall	0.954/0.953/0.953	0.933/0.931/0.931	0.963/0.962/0.962

### 4.2 Performance of standalone NER module

Table 4 presents the number of P, I, C, and O elements in three datasets. Table 5 and Supplementary Results Table S5 show the performance of PubMedBERT on PICO extraction (i.e. token-level and entity-level respectively) on all three datasets. We observed that our pipeline achieves higher performance on AD and COVID-19 datasets than the EBM-NLP_*mod*_ dataset. Our approach looks promising with F1 score >0.8 for token-level evaluation and >0.68 for entity-level evaluation on all three datasets.

**Table 4.** Statistics of PICO elements in all three corpora.

	P	I	C	O	Total	Docs
EBM-NLP_*mod*_	529	1657	240	1337	3763	500
COVID-19	262	602	180	603	1647	150
AD	215	467	103	626	1411	150
Total	1006	2726	523	2566	6821	800

**Table 5.** Entity-level evaluation of the standalone NER model on all three corpora by exact match.

	EBM-NLP_*mod*_	COVID-19	AD
Entity	P/R/F1	P/R/F1	P/R/F1
P	0.752/0.778/0.765	0.868/0.882/0.875	0.801/0.823/0.812
I	0.649/0.682/0.665	0.820/0.857/0.838	0.768/0.792/0.780
C	0.650/0.571/0.608	0.780/0.750/0.765	0.766/0.796/0.781
O	0.683/0.691/0.687	0.827/0.857/0.842	0.792/0.813/0.802
Overall	0.676/0.692/0.683	0.826/0.849/0.838	0.783/0.807/0.795

### 4.3 End-to-end evaluation of PICO extraction system

The token-level F1 score for the EBM-NLP_*mod*_ dataset, COVID-19 dataset, and AD dataset, was 0.833, 0.928, and 0.899. Similarly, the entity-level F1 score for these datasets was 0.712, 0.850, and 0.805. The performance of our two-step NLP pipeline system is better across all datasets when compared to the standalone NER module.

## 5 Discussion and future work

Our section-specific annotation schema aimed to reduce annotation inconsistencies by classifying sentences before NER, decreasing annotation complexity and time. Although focused on the methods section, it can be extended to other sections if needed. Our method balances minimizing complexity and information loss, covering 95.2% of all entities in the Methods section and improving inter-annotator agreement. Our system has significantly enhanced PICO extraction performance, but with a modest impact on the COVID-19 dataset, possibly due to its specificity and contemporary nature.

However, there is further scope for improvement. To perform error analysis, we evaluated our NER results on the entity-level by partial match. The models achieved 0.848, 0.924, and 0.899 for EBM-NLP_*mod*_, COVID-19, and AD datasets, respectively. Detailed performance by different entity types and the confusion matrix are shown in Supplementary Results Table S7 and Supplementary Results Fig. S2. The F1 score for the Control element is lower when compared to other elements across all three datasets (Tables 5 and 6). The confusion matrix shows that 17% of controls are confused with the Intervention element of PICO. In several studies, Control and Intervention elements are the same and it is difficult to distinguish them without a proper context. In most cases, a proper understanding of whether an intervention was applied to a “study group” or a “control group” is required to distinguish between the Control and Intervention elements. The sentence, “Patients with severe COVID-19 were randomly divided into two groups: the standard treatment group and the standard treatment plus hUC-MSC infusion group,” says that the standard treatment was given to both groups. The first mention was annotated as the control and the second mention was annotated as the Intervention element. This may be confusing to the model because of the lack of context to learn the difference between the Intervention and Control elements. To ameliorate this confusion between Intervention and Control elements, we need to strategically incorporate more contextual information. This might be achieved by performing NER on all the sentences from each abstract together, rather than processing each sentence in isolation. This approach has the potential to provide the model with richer contextual information. We also observed that numerous Control elements labeled as “Placebo,” lead to model overfitting. For instance, in the sentence, “either 3 weeks of taper and 5 weeks of placebo only or continuing use of risperidone.” The Control is “5 weeks of placebo” as per the annotation guidelines. However, the model only partially identified “placebo” as the Control. Such issues can be resolved by incorporating more specific annotation rules, such as, “do not include any prepositional phrases preceding a Control element.”

**Table 6.** An end-to-end entity-level evaluation on all three datasets.

	EBM-NLP_*mod*_	COVID-19	AD
Class	P/R/F1	P/R/F1	P/R/F1
BG	0.744/0.778/0.760	0.874/0.870/0.872	0.793/0.813/0.803
MT	0.683/0.695/0.689	0.827/0.841/0.834	0.765/0.800/0.783
RS	0.705/0.664/0.684	0.790/0.794/0.792	0.748/0.819/0.782
CC	0.724/0.728/0.726	0.861/0.887/0.874	0.811/0.844/0.827
Overall	0.708/0.716/0.712	0.843/0.857/0.850	0.789/0.823/0.805

Nearly 19% of the “Inside” label was misclassified as the “Outside” label in the Participant element. But the “Beginning” label of the Participant element is well-identified. This suggests that the NER model struggles with discerning the ending position of the Participant element. The issue may arise from our annotation guidelines, which include the rule for annotating the longest noun phrase for the Participant element. Clear boundary rules might address this. For example, we could stipulate that “only one prepositional phrase in a Participant element should be included.”

The overall performance of our PICO extraction system also depends on the performance of the sentence classification model. While our sentence classification model categorizes the sentences into general categories based on rhetorical roles, this could be extended to classifying sentences directly into P, I, C, and O categories. The NICTA-PIBOSO corpus incorporated P, I, and O categories in addition to the background and study design, with every sentence belonging to a single category. However, it is very common that a sentence may include multiple PICO elements. Hence, developing a multi-label sentence classification model for identifying PICO categories will be more beneficial when compared to the existing classification model.

The broader applicability of our approach is yet to be established. Testing models trained on specific datasets like AD and COVID-19 on other diseases may offer insights. Utilizing transfer learning could save further annotation time.

Many existing studies (Nye *et al.* 2018, Zhang *et al.* 2020) including our work use only abstracts to identify the PICO elements from PubMed articles. However, certain interventions and outcomes are reported only in the full-text of the article. Our approach will miss extracting the interventions and outcomes mentioned only in the full-text articles. Developing a system that can extract PICO elements from both PubMed abstract and full-text articles or applying our pipeline to the full-text might be useful.

In the future, we plan to develop an interface that can take a PubMed identifier or an abstract as input and returns a list of extracted PICO elements as output. The Cochrane Review currently provides a PICO search engine associated with their reviews. However, those PICO elements are manually curated from a limited number of articles.

## 6 Conclusions

We presented a new two-step extraction approach to extract PICO elements from the RCT abstracts. We modified annotation guidelines to improve the annotation quality and IAA and reduce annotation complexity for PICO extraction. By annotating the method section and title alone, we not only reduce annotation complexity for PICO extraction but were able to achieve a much higher performance on retrieving unique PICO elements without much loss of information from a subset of RCT abstracts from the EBM-NLP corpus. We verified the usability and reliability of our system by applying and evaluating it on an unseen dataset.

## Supplementary Material

btad542_Supplementary_Data
