# Supplementary material for: Towards precise PICO extraction from abstracts of randomized controlled trials using a section-specific learning approach
Source: Bioinformatics. 2023 Sep 5;39(9):btad542. doi: 10.1093/bioinformatics/btad542 (PMC10500081; doi:10.1093/bioinformatics/btad542)
Supplement: btad542_Supplementary_Data [file btad542_supplementary_data.docx]

# Supplementary Information:

**Towards precise PICO extraction from abstracts of randomized controlled trials using a section-specific learning approach**

Yan Hu^1^, Vipina K. Keloth^2^, Kalpana Raja^2^ and Hua Xu^2,*^

^1^ School of Biomedical Informatics, University of Texas Health Science Center at Houston, Texas, USA.

^2^ Section of Biomedical Informatics and Data Science, School of Medicine, Yale University, Connecticut, USA.

*To whom correspondence should be addressed.

**1. Supplementary Methods**

**1.1 Prompt-based learning for sentence classification**

Traditional sentence classification methods usually involve a two-step process: encoding the input sentence into a fixed-size vector representation (using models like LSTM, CNN, or Transformer) [1,2,3], and then decoding this vector into the class labels (using a softmax function over the labels) [4]. Prompt-based learning simplifies this process by directly mapping the input to the output using natural language prompts [5]. In our previous study, we delved into the adaptation of prompt-based learning for sentence classification tasks within RCT abstracts by applying 5 different prompt designs in combination with PubMedBERT. The most effective prompt appeared to be the one that inserted a mask prior to the sentence which needs to be classified (refer to Table S2 below for a visual explanation). Using PMID 31872515 as an example, Table S1 demonstrates sentences (right column) and their associated sections (left column).

**Table S1.** An example of Section to Sentence Correspondence by PMID 31872515.

| **Section name** | **Sentence** |
| --- | --- |
| OBJECTIVE | To investigate the optimal … |
| BACKGROUND | Diabetic patients are at high risk … |
| METHODS | This post hoc study analyzed … |
| RESULTS | In total, 1,773 diabetic … |
| CONCLUSIONS | Premature DAPT discontinuation … |

**Table S2.** The prompt format and its corresponding input examples to the model. In this prompt format, {Mask} and {Sentence} are two variables.

| **Prompt** |
| --- |
| {Sentence 1} {Sentence 2} ... {Mask n}: {Sentence n} |
| **Examples** |
| {OBJECTIVE}: To investigate the optimal dual… Diabetic patients are at … |
| To investigate the optimal dual… {BACKGROUND}: Diabetic patients are at… |

The variable {Mask n} represents the section corresponding to {Sentence n}. Using PMID 31872515 as an example, {Mask 1} stands for {OBJECTIVE} and {Sentence 1} equates to {To investigate the optimal dual…}. Similarly, {Mask 2} stands for {BACKGROUND} and {Sentence 2} matches {Diabetic patients are at…}.

During the training phase, the model was provided with the section names, but these were deliberately masked during prediction. This approach aligns with one of the key pretraining objectives of BERT models, specifically, predicting randomly masked tokens within a sentence. The primary benefit of this method is that it maximizes the use of knowledge acquired during the pretraining process.

**1.2 Inter-annotator agreement**

Cohen's kappa is a statistical measure used to evaluate inter-annotator agreement or reliability for categorical data, including NER tasks. It takes into account the agreement between two or more annotators beyond what would be expected by chance alone. The formula for Cohen's kappa is as follows:

$$kappa= \frac{P_{o}-P_{e}}{1-P_{e}}$$

where:

Po is the observed agreement, which is the proportion of instances where the annotators agreed.

Pe is the expected agreement by chance, which represents the agreement that would be expected due to random chance.

To calculate P_o_ and P_e_:

$$P_{o}= \frac{A}{N}$$

$$P_{e}=\sum_{i=1}^{n} (\frac{\sum_{j=1}^{n} A_{i}\times B_{j}}{N^{2}})$$

where:

A the number of instances where their annotations match.

N is the total number of instances or entities.

n is the number of categories or labels

Ai is the sum of the annotations for category i by annotator A.

Bj is the sum of the annotations for category j by annotator B.

**1.3  NER evaluation**

For each Entity type:

$$Precision =\frac{TP}{TP+FP}$$

$$Recall=\frac{TP}{TP+FN}$$

$$F1=\frac{2*Precision*Recall}{Precision+Recall}$$

Where:

TP signifies True Positives.

FP indicates False Positives.

FN represents False Negatives.

For Micro Averaged scores:

$${Precision}_{micro}=\frac{\sum_{i=1}^{n} {TP}_{i}}{\sum_{i=1}^{n} {TP}_{i}+\sum_{i=1}^{n} {FP}_{i}}$$

$${Recall}_{micro}=\frac{\sum_{i=1}^{n} {TP}_{i}}{\sum_{i=1}^{n} {TP}_{i}+\sum_{i=1}^{n} {FN}_{i}}$$

$$F1=\frac{2*{Precision}_{micro}*{Recall}_{micro}}{{Precision}_{micro}+{Recall}_{micro}}$$

Where:

 n is the number of classes.

**References:**

1. Hochreiter, Sepp, and Jürgen Schmidhuber. "Long short-term memory." *Neural computation* 9.8 (1997): 1735-1780.
2. Chen, Yahui. *Convolutional neural network for sentence classification*. MS thesis. University of Waterloo, 2015.
3. Vaswani, Ashish, et al. "Attention is all you need." *Advances in neural information processing systems* 30 (2017).
4. Bridle, John S. "Probabilistic interpretation of feedforward classification network outputs, with relationships to statistical pattern recognition." *Neurocomputing: Algorithms, architectures and applications*. Springer Berlin Heidelberg, 1990.
5. Brown, Tom, et al. "Language models are few-shot learners." *Advances in neural information processing systems* 33 (2020): 1877-1901.

**2.         Supplementary results**

**Table S3.** Performance of different models on the original EBM-NLP corpus evaluated on token-level and entity-level

| Models | Precision | Recall | F1 |
| --- | --- | --- | --- |
| LSTM-CRF (token-level) | 0.68 | 0.66 | 0.67 |
| LSTM-CRF (entity-level) | 0.49 | 0.32 | 0.39 |
| PubMedBERT (token-level) | 0.73 | 0.73 | 0.73 |
| PubMedBERT (entity-level) | 0.53 | 0.39 | 0.45 |

**Table S4.** The performance comparison of prompt-learning-based and HSLN-based sentence classification

|  | Prompt-learning | | |  | HSLN | | |
| --- | --- | --- | --- | --- | --- | --- | --- |
|  | EBM-NLP*_mod_* | COVID-19 | AD |  | EBM-NLP*_mod_* | COVID-19 | AD |
| Class | P/R/F1 | P/R/F1 | P/R/F1 |  | P/R/F1 | P/R/F1 | P/R/F1 |
| BG | 0.911/0.977/0.943 | 0.897/0.975/0.934 | 0.934/0.988/0.960 |  | 0.969/0.946/0.958 | 0.873/0.955/0.912 | 0.952/0.954/0.953 |
| MT | 0.946/0.952/0.949 | 0.913/0.933/0.923 | 0.956/0.953/0.955 |  | 0.931/0.904/0.917 | 0.887/0.932/0.909 | 0.874/0.961/0.916 |
| RS | 0.967/0.961/0.964 | 0.977/0.887/0.930 | 0.987/0.948/0.967 |  | 0.932/0.977/0.954 | 0.916/0.925/0.920 | 0.952/0.955/0.953 |
| CC | 0.989/0.910/0.948 | 0.947/0.955/0.951 | 0.965/0.975/0.970 |  | 0.956/0.939/0.947 | 0.974/0.876/0.922 | 0.965/0.912/0.938 |
| Overall | 0.954/0.953/0.953 | 0.933/0.931/0.931 | 0.963/0.962/0.962 |  | 0.947/0.947/0.947 | 0.920/0.918/0.918 | 0.944/0.943/0.943 |

**Table S5.**Token-level evaluation of the standalone NER model on all three datasets.

|  | EBM-NLP*_mod_* | COVID-19 | AD |
| --- | --- | --- | --- |
| Entity | P/R/F1 | P/R/F1 | P/R/F1 |
| P | 0.868/0.851/0.885 | 0.942/0.942/0.942 | 0.899/0.864/0.937 |
| I | 0.749/0.753/0.746 | 0.863/0.884/0.843 | 0.847/0.864/0.830 |
| C | 0.630/0.559/0.722 | 0.799/0.791/0.806 | 0.777/0.784/0.771 |
| O | 0.781/0.775/0.787 | 0.931/0.939/0.923 | 0.903/0.915/0.892 |
| Overall | 0.793/0.784/0.802 | 0.917/0.923/0.911 | 0.888/0.884/0.892 |

**Table S6.**An end-to-end token-level evaluation on all three datasets

|  | EBM-NLP*_mod_* | COVID-19 | AD |
| --- | --- | --- | --- |
| Class | P/R/F1 | P/R/F1 | P/R/F1 |
| BG | 0.858/0.842/0.874 | 0.951/0.941/0.961 | 0.894/0.879/0.910 |
| MT | 0.783/0.775/0.792 | 0.873/0.877/0.869 | 0.840/0.853/0.828 |
| RS | 0.724/0.678/0.778 | 0.821/0.838/0.805 | 0.792/0.814/0.771 |
| CC | 0.832/0.824/0.840 | 0.945/0.953/0.938 | 0.929/0.929/0.930 |
| Overall | 0.820/0.808/0.833 | 0.928/0.927/0.928 | 0.897/0.894/0.899 |

**Table S7.** Entity-level evaluation of the PubMedBERT model on all three corpora by partial match.

|  | EBM-NLP*_mod_* | COVID-19 | AD |
| --- | --- | --- | --- |
| Entity | P/R/F1 | P/R/F1 | P/R/F1 |
| P | 0.883/0.917/0.899 | 0.973/0.958/0.966 | 0.923/0.936/0.929 |
| I | 0.827/0.825/0.826 | 0.905/0.918/0.911 | 0.857/0.883/0.870 |
| C | 0.826/0.773/0.799 | 0.840/0.850/0.845 | 0.816/0.894/0.853 |
| O | 0.863/0.866/0.865 | 0.934/0.958/0.946 | 0.917/0.929/0.923 |
| Overall | 0.847/0.849/0.848 | 0.918/0.931/0.924 | 0.886/0.912/0.899 |


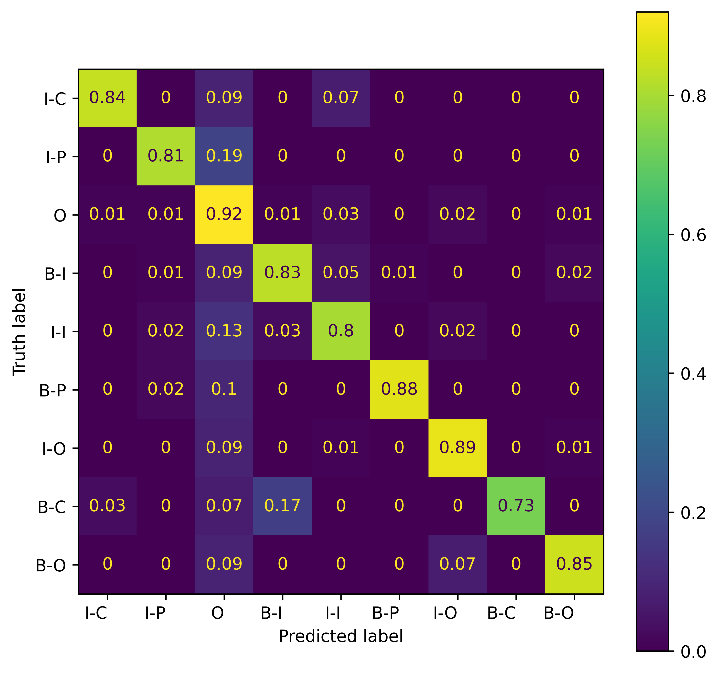


**Fig. S1.**Normalized confusion matrix of the prediction results from EBM- NLP*_mod_*

**3.         Annotation guidelines**

**Definitions:**

| Participant (P) | The "P" component refers to the descriptions of participants involved in a medical study, including details about their recruitment process and the characteristics or requirements they needed to meet for inclusion. These descriptions typically encompass various relevant factors such as age, gender, sample size, medical diagnoses or conditions, treatment locations, and other specific details pertinent to the study being conducted. These population descriptors provide important context and help define the target group under investigation. |
| --- | --- |
| Intervention (I) | The "I" component represents the intervention, which refers to the proposed treatment or approach being administered to the participants. While interventions commonly refer to medical treatments in the medical literature, it's important to note that interventions can also encompass non-medical approaches, such as educational courses or musical therapies. The intervention is the specific action or method being implemented with the aim of addressing or influencing the condition or problem being studied. |
| Control (C) | The "C" component refers to the comparison or control treatment utilized in many studies. The control group serves as a baseline for comparison to evaluate the effectiveness of the intervention in terms of the desired outcomes. In some cases, the control group may receive a placebo treatment, which is an inactive substance or sham procedure that mimics the appearance of the actual intervention but lacks the active components. Alternatively, the control group may receive no treatment at all. These control treatments are implemented to provide a reference point for assessing the impact of the intervention and determining its efficacy in relation to the desired outcomes. |
| Outcome (O) | The "O" component refers to outcomes, which represent the measurements or observations used to assess the effectiveness of the treatment in individuals participating in a trial. Outcomes are often described by specifying the specific score, scale, measurement tool, or clinical test utilized to evaluate the desired outcome. In clinical trials, researchers compare outcomes between two or more groups of patients, each receiving a different treatment. These outcomes serve as measurable indicators to determine whether the treatment has produced the intended effect or achieved the desired result. By comparing outcomes across different treatment groups, researchers can assess the comparative effectiveness of the interventions being studied. |

**ALL THE PICO ELEMENTS SHOULD FOLLOW GENERAL RULES.**

**SPECIFIC RULES ONLY APPLY TO PARTICULAR ELEMENT AS STATED.**

**All the examples in blue font should be included while those in red should be avoided.**

**General Rules:**

1.   Always include the article words before the entities - ‘the’, ‘a’, ‘an’.

*Example: with 33 randomized to the face-to-face teaching*

All the markable concepts should be derived from noun phrases.

2.  Include special characters.

**What sections need to be annotated:**

1.   Title

2.   Methods section

**Specific Rule:**

*Participant:*

What to include:

1. Mark the longest noun phrases about how the participants were recruited in the medical study and what characteristics or requirements they needed to meet to be included in the study.

It is usually described as: *XX patients with XXX were recruited.*

*Example:Patients with the confirmed COVID-19 based on the PCR test are eligible to participate in the trial if they are 18 to 75 years of age and have no history of the current use of warfarin or propolis supplement and presence of sensitivity to bee products.*

*Example: Effects of a leisure programme on quality of life and stress of individuals with ASD.*

*Example: Superior survival with capecitabine plus docetaxel combination therapy in anthracycline-pretreated patients with advanced breast cancer*

*Example: A random sample of 60 eligible patients with back pain (28 men, 32 women) were recruited by general practitioners and self-referral for physiotherapy treatment and randomly assigned to 1 of 3 groups.*

*Example: Dual-task-related gait changes in transitionally frail older adult*

1. When the subgroup information is inevitably included in the complete noun phrase, include it.

*Example: The study population included 403 of the original 479 patients in the trial who had completed six days of blinded therapy without refractory angina or myocardial infarction*.

What to exclude:

1. Phrases about grouping should not be considered as P

*Example: The study encompasses a wide range of COPD patients, e.g. patients with stable cardiac diseases including arrhythmia can be included.*

1. When a location is mentioned together with participants, include them. Otherwise, do not include them.
2. Do not annotate inclusion/exclusion criteria for the participants when the phrases “inclusion criteria” or “exclusion criteria” are explicitly mentioned.
3. Do not include phrases that only mention a condition without participants being mentioned

*Example: Visual Assessment of Relative Apical Sparing Pattern Is More Useful Than Quantitative Assessment for Diagnosing Cardiac Amyloidosis in Borderline or Mildly Increased Left Ventricular Wall Thickness*

1. Exclude prepositional words before the participants

*Example: In eight healthy volunteers (seven males; mean age 32 years)*

*Intervention:*

What to include:

1. Include all the modifiers before the I element, when dosage, frequency, and the form of the intervention are as modifiers.

*Example: high-dose vaccine (intervention), low-dose vaccine (control)*

*All patients had received monthly leuprolide acetate*

1. Include an I entity when it is in a prepositional phrase

*Example: Patients were randomized to a high dose of vaccine group and …..*

1. If there are multiple distinct entities mentioned within the same context, they should be split into separate annotations.

*Example1: Perioperative blood transfusions, with or without allogeneic leucocytes, relate to survival, not to cancer recurrence.*

*Example2: 49 patients who underwent gastrectomy with Longmire-Interposition with (n = 33) or without (n = 16) an additional pouch and 46 patients with a Roux-en-Y-reconstruction were analysed retrospectively.*

*Example3: to have the DFT determined using biphasic shocks at two durations of VF each (5 and 10 s, 10 and 20 s or 5 and 20 s)*

*Example: self-administered either 0.005%, 0.01%, 0.05% P-113 or placebo mouthrinse formulations twice daily over a four-week treatment period.*

1. Annotate continuous entities separately

*Example1: were randomized to five ad libitum diets for 12 months: high P/LGI (HP/LGI)*

*Example2： Long-term follow-up of a randomized trial of fludarabine-mitoxantrone, compared with cyclophosphamide, doxorubicin, vindesine, prednisone (CHVP)*

1. Include all the general and specific terms of intervention

*Example: Title: Impact of different platelet glycoprotein IIb/IIIa receptor inhibitors among diabetic patients undergoing percutaneous coronary intervention:*

1. Annotate intervention every time it appears no matter it has been annotated or not

*Example: A-daily placebo; B-daily norethindrone acetate 5 mg; C-daily norethindrone acetate 5 mg and conjugated equine estrogens 0.625 mg;*

1. In case one drug is co-administered with another one, annotate them separately.

*Example: a sulfonylurea when coadministered with an ACE inhibitor*

What to exclude:

1. Do not include general terms like ‘*control group*’ or  ‘*intervention group’, “a treated* group”

1. When intervention is mentioned as a group, only annotate the intervention itself. Do not include the word ‘group’

*Example: with 33 randomized to the face-to-face teaching group (control group) and 29 to the WSSL group(case group)*

1. Exclude dosage, frequency, length, and the form of the intervention, when they are located after the I element.

*Example: XXX drug 10 mg …*

1. Do not include any prepositional phrase following the I element.

*Example: low-intensity exercise once a week*

*Control:*

1. All the treatments/procedures that are given to all the patients in the control group should be annotated as control

1. C entities follow exactly the same rules as I entities. The only difference is that C is semantically the comparator treatment.

1. C and I sometimes could be the same thing, like:

*Example: The study group received NET and personalized psychological intervention, while the control group only received personalized psychological intervention.*

In these cases, annotate them according to semantics. In the above case, the 1st *personalized psychological intervention* should be ‘I’ and 2nd *personalized psychological intervention* is ‘C’.

*Outcome:*

What to include:

1. If a Clinical test is the main part of the study and is being used as an intervention, then it has to be annotated as “I”.

1. Include the outcomes separately when their abbreviations and expansion are mentioned

*Example: Main outcomes: The primary endpoint is the improvement of oxygenation as measured by mean and/or median change from pre-treatment (day 1) to post-treatment (day 6 and 15 or at discharge, whichever comes first) in PaO2/FiO2 ratio, P(A-a)O2 gradient and a/A PO2 ratio.* (*PAO2=* *Partial alveolar pressure of oxygen, PaO2=partial arterial pressure of oxygen, FiO2=Fraction of inspired oxygen*).

1. Include an outcome that is illustrated both at a summative level and detailed level

1. Include up to one prepositional phrase

1. If an affirmative sentence is provided, then the outcome should be what suggests the result of the study.

*Example: The efficacy of sublingual immunotherapy for respiratory allergy is not affected by different dosage regimens in the induction phase.*

1. Include words indicating the statistical measurement like “log” “mean” and “proportion” before the outcome

*Example: Mean changes in log serum level of PSA*

*Example: The study was designed to demonstrate a difference in the proportion of women with clear histological margins of 82% for LLETZ*

1. Include the words “a composite of ” when two outcomes are combined

*Example:  The primary end point is a composite of incident* *or recurrent CVD outcomes, that is, coronary heart, cerebrovascular, or abdominal aortic/lower extremity arterial events.*

What to exclude:

1. Do not include how the outcome was measured unless it is a modifier

*Example: a validated questionnaire on self reported measures*

1. Do not include the time span or time point where the outcome was measured, unless it is a modifier, or the measurement depends on time

*Example: The primary outcome is the change in MMSE from the beginning of the trial…*

1. Do not include how often the outcome was measured, unless it is a modifier

*Example: The primary outcome is the change in a 12-week MMSE from the beginning of the trial…*

1. Do not include who received the outcome measure

*Example: The primary outcome is the change in MMSE in Mild-to-moderate dementia patients …*

1. Do not include information about how an outcome is compared

*Example: The primary outcome is the difference in MMSE compared to the healthy participant*

1. Do not include the word that indicates the difference like “change”, “difference”

*Example: The primary outcome is the change in MMSE …or their effect on mortality,*

*Example: To detect trends* *in additional imaging recommendations*

1. Do not include statistical methods

*Example: using Cox proportional hazards regression analysis of data collected from 6,781 patients randomized into the Studies of Left Ventricular Dysfunction trials*

*Example: MANCOVA, ANCOVA and Post Hoc analyses* *were utilized to test for significant differences among the three groups.*

1. Do not annotate outcomes in titles
